# Cytomegalovirus Enterocolitis in a Patient Treated with Methylprednisolone for Amiodarone Hypersensitivity Pneumonitis

**DOI:** 10.3390/diagnostics14232633

**Published:** 2024-11-22

**Authors:** Darinka Purg, Marko Hojnik, Nika Aleksandra Kravos Tramšek

**Affiliations:** 1 Department of Gastroenterology, University Medical Centre Maribor, Ljubljanska Ulica 5, 2000 Maribor, Slovenia; darinka.purg@ukc-mb.si; 2 Department of Pathology, University Medical Centre Maribor, Ljubljanska Ulica 5, 2000 Maribor, Slovenia; marko.hojnik@ukc-mb.si; 3 Department of Endocrinology and Diabetology, University Medical Centre Maribor, Ljubljanska Ulica 5, 2000 Maribor, Slovenia

**Keywords:** cytomegalovirus enterocolitis, immunodeficiency, amiodarone pneumonitis, methylprednisolone

## Abstract

Cytomegalovirus (CMV) is a common cause of infection in immunocompromised individuals, such as patients with hematological malignancies or AIDS, but can also occur in patients with other acquired immunodeficiencies. In tissue-invasive diseases, CMV diagnosis requires CMV DNA in the plasma and the histological confirmation of CMV in a tissue or organ. Evidence of CMV colitis requires a characteristic endoscopic picture with ulcers with a well-defined, convex appearance and CMV viral inclusions in the form of an “owl’s eye” on mucosal sections stained with hematoxylin and eosin. CMV-specific immunohistochemistry is the gold standard for identifying CMV in tissue biopsies. It is important to consider a CMV infection in the diagnostic process, as it may delay the diagnosis and the treatment. We present the case of a 78-year-old patient with amiodarone interstitial lung disease who was treated with methylprednisolone. Two weeks after the start of his treatment, he was admitted to the hospital for acute gastroenterocolitis and Addisonian crisis. An examination had confirmed a tissue-invasive CMV disease. He was treated with valganciclovir for a total of six weeks. After the completion of treatment, the patient showed no clinical signs of CMV infection, and both laboratory and histological examinations revealed no residual CMV disease. Tissue-invasive CMV disease can occur in patients with acquired immunodeficiency, which may result from various causes, including glucocorticoid treatment.

**Figure 1:**
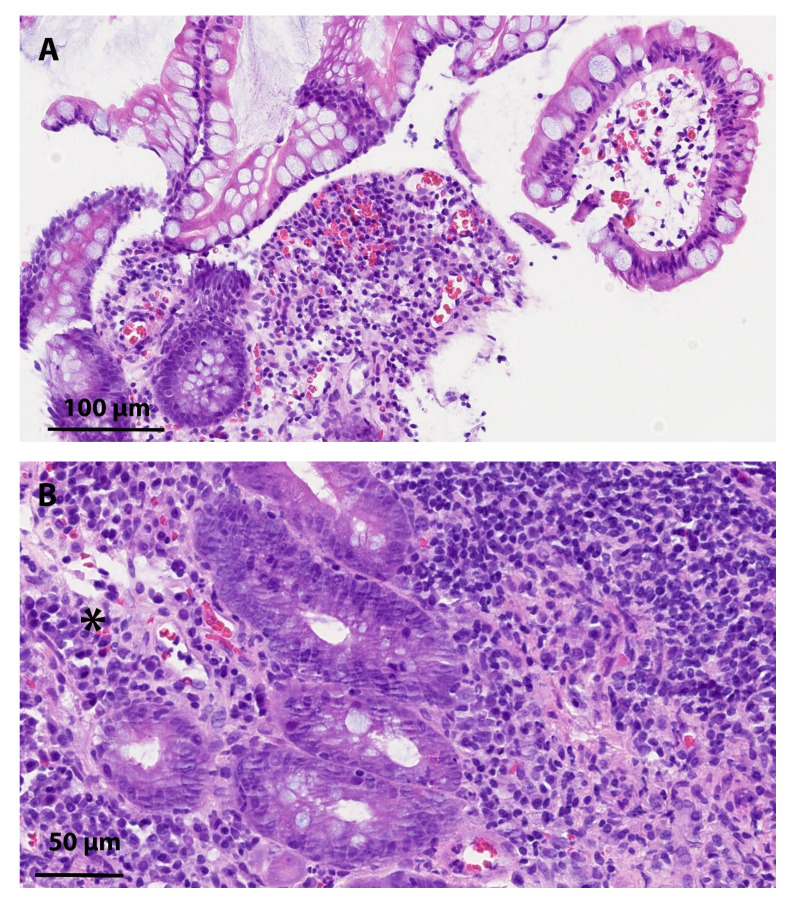

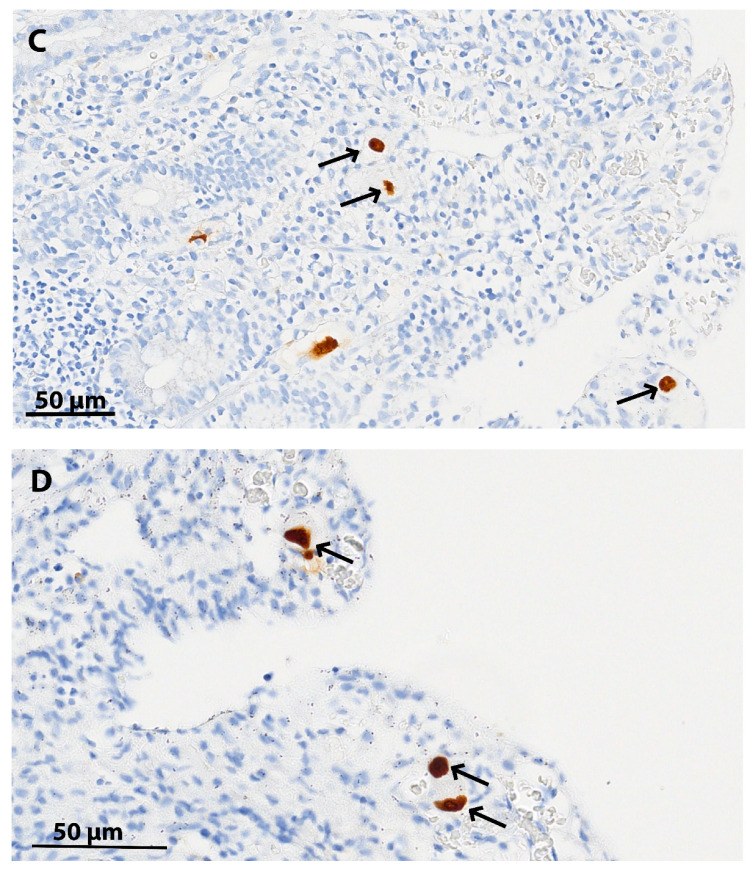
(**A**–**D**): Hematoxylin and eosin staining (**A**,**B**) and immunohistochemical CMV staining (**C**,**D**) of the patient’s tissue samples from terminal ileum. Severe tissue-invasive CMV disease of the gastrointestinal tract and even disseminated CMV disease can occur in immunocompromised patients. Gastrointestinal infections remain a common cause of mortality in these patients [1]. The typical histological findings of CMV infection are the presence of intranuclear “owl’s eye” inclusions in the endothelial and mesenchymal cells, thickened nuclear membrane, coarse red intracytoplasmic granules, increased apoptotic bodies, an inflammatory infiltrate (mixed inflammatory plasma cell rich infiltrate) in mucosa, often accompanied with an area of necrosis and ulcerations [2]. We present a 78-year-old Caucasian male patient, with a history of nonischemic dilatative cardiomyopathy with reduced left ventricular ejection fraction (20%), asthma, and type 2 diabetes mellitus. He had no previously known gastrointestinal medical conditions, except for having undergone surgery for an inguinal hernia as a young adult. A cardiologist introduced amiodarone treatment for the past four years to prevent another episode of atrial fibrillation. He has also been receiving furosemide, bisoprolol, acetylsalicylic acid, rosuvastatin, and a combination of inhalation therapy (formoterol, beclomethasone, and glycopyrronium bromide). In the last three years, his chronic conditions had been well managed, and he had not required hospitalization until recently. Three weeks prior to the diagnosis of the CMV infection, he was hospitalized due to worsening community-acquired bacterial pneumonia with increasing respiratory insufficiency. However, a high-resolution computed tomography (HRCT) of the chest showed typical changes related to amiodarone-induced interstitial pneumonitis. Bronchoscopy and biopsy were not considered due to comorbidities and the typical HRCT findings. Amiodarone was discontinued, and the methylprednisolone therapy was initiated at a dose of 0.5 mg/kg body weight and pantoprazole 40 mg OD during the whole time of methylprednisolone treatment. After 14 days of treatment with methylprednisolone, both diarrhea and vomiting occurred. He defecated up to 10 times per day but had no fever. On day 18, he was admitted to the hospital due to hypotension and tachycardia, which were recognized as an Addisonian crisis with etiologically nonspecific diarrhea. Laboratory findings included a mild normocytic anemia, a mild hypokalemia, transient deterioration of renal function, elevated C-reactive protein, decreased levels of total calcium, vitamin D, and fibrinogen, hypoalbuminemia, and a rise in INR without anticoagulation therapy. Microbiological specimens were collected, and ceftriaxone was empirically administered. Our patient required hydrocortisone replacement therapy. A buccal swab confirmed Herpes simplex virus type 1 gingivostomatitis and a positive serum CMV DNA level (372 IU/mL). Valacyclovir was started orally to treat stomatitis for a period of five days. Due to the positive CMV DNA and persistence of diarrhea, further diagnostics were performed. On an abdominal CT scan, we found signs of infectious enteritis (Figure 2A,B). An upper gastrointestinal endoscopy confirmed esophageal candidiasis and altered duodenal mucosa. Histology showed mild to moderate Helicobacter pylori chronic gastritis without glandular atrophy, intestinal metaplasia or intraepithelial neoplasia, and mild chronic duodenitis. No histopathological changes were suggestive of the chronic inflammatory bowel disease, coeliac disease, parasitic infection, Whipple’s disease or *Giardia* spp. Inflammatory lesions with multiple ulcers in the terminal ileum and a rectal ulcer were seen on colonoscopy with terminal ileoscopy. Histology confirmed the presence of CMV in the terminal ileum (**A**–**D**) and in the random samples of the colonic mucosa. We repeated serum CMV DNA. The viral load was high (44,900 IU/mL), along with the presence of CMV specific IgG and IgM. We confirmed the presence of tissue-invasive CMV disease in an immunocompromised patient. He was treated with valganciclovir at a dose of 900 mg BID orally. After seven days of treatment, a decline in viral load (serum CMV DNA 2380 IU/mL) was observed. We continued treatment on an outpatient basis as the clinical condition improved with relief of the intestinal syndrome. All other comorbidities were in a stable phase, and we continued methylprednisolone therapy at a low dose (4 mg daily). The CMV DNA was regularly checked and, according to the results, valganciclovir therapy was discontinued after six weeks. Follow–up endoscopy examinations and histological mucosal samples showed no signs of CMV disease. This Figure shows small intestinal mucosa biopsy samples in the patient with CMV infection. The villi were normally dense with epithelium without atypia. The lamina propria was focally mildly fibrosed, moderately infiltrated with mononuclear cell inflammatory infiltrate rich with plasma cells ((**B**)—area with symbol *). A few eosinophilic and neutrophilic granulocytes were also present in the inflammatory infiltrate. Lymphatic aggregates were present. There were no signs of cryptitis, no crypt microabscesses and no epithelioid granulomas. Immunohistochemical staining revealed nuclear staining in CMV infected cells ((**C**,**D**)—marked with arrows). Paraffin-embedded tissue blocks were sectioned with a microtome to obtain 3–5 μm-thick paraffin sections and were placed onto a glass slide (StarFrost; Knittel, Braunschweig, Germany). Hematoxylin and eosin staining was performed on a fully automated VENTANA HE 600 system according to standard protocol. Immunohistochemical staining was performed for the visualization and localization of CMV specific antigens on formalin-fixed, paraffin-embedded tissue samples from terminal ileum. Paraffin-embedded tissue blocks were sectioned with a microtome to obtain 3–5 μm-thick paraffin sections, which were placed onto a glass slide (Superfrost Plus; Epredia, Portsmouth, UK). The tissue slides were dehydrated in a slide-drying ventilation oven for 60 min at 60 °C. IHC staining was carried out on an automated system (BenchMark Ultra; Roche Tissue Diagnostics’, Mannheim, Germany) using detection kits (UltraView Universal DAB detection kit; Roche Tissue Diagnostics’, Mannheim, Germany; cat. no. 05269806001) following the manufacturer’s instructions. A deparaffinization solution (EZPrep solution; Roche Tissue Diagnostics’, Mannheim, Germany; cat. no. 05279771001) was used for 4 min at 72 °C for the complete dissolution of the paraffin. Protease (Protease 1; Roche Tissue Diagnostics’, Mannheim, Germany; cat. no 05266688001) was used for the epitope retrieval for 8 min. The slides were applied and incubated with the primary antibodies anti-CMV (Sigma-Aldrich; Cell Marque, Rocklin, CA, USA; cat. no. 213M-16-RUO, lot: 0000285872) for 24 min at 36 °C (optimized dilution 1:100 in an antibody diluent (Ventana; Roche Tissue Diagnostics’, Mannheim, Germany; cat. no. 05261899001)). The specific anti-CMV antibody was located by a specific secondary antibody to which an enzyme-HRP labelled tertiary antibody was bound. The complex was then visualized with hydrogen peroxide substrate and 3,3′-diaminobenzidine tetrahydrochloride (DAB) chromogen, which produces a brown precipitate that was visible by light microscopy. CMV infection may manifest as the CMV syndrome (fever, malaise, myalgia, etc.) or as tissue-invasive CMV disease. It can affect the gastrointestinal tract from the oral cavity to the anus [3,4]. Detecting the CMV-specific IgM and IgG antibodies demonstrates a primary CMV infection. In immunocompromised patients with clinical signs, the diagnosis of CMV disease requires the presence of CMV DNA in the plasma and histological confirmation of CMV in a tissue or an organ [5]. CMV-specific immunohistochemistry (IHR) is the gold standard for identifying CMV in tissue biopsies [6]. The data showed that CMV infection was detected more frequently if the referring physician requested an IHR for the CMV when the patient was immunocompromised due to either underlying disease or immunosuppressive drugs, if more biopsy specimens were obtained, or if the specimens showed severe inflammation [7,8]. At our center, immunohistochemical staining for CMV is performed either at the clinician’s request for immunocompromised patients or at the pathologist’s request due to microscopic features on biopsy, including the degree and type of inflammation and viral cytopathic effects. The most common manifestation of invasive CMV disease is gastrointestinal involvement. Evidence of CMV colitis requires a characteristic endoscopic picture with ulcers with a well-defined, convex appearance and CMV viral inclusions in the form of an “owl’s eye” on mucosal sections stained with hematoxylin and eosin [4]. Treatment can be challenging as viremia may be low or even absent despite a marked clinical picture of gastrointestinal involvement. Relapses can also be expected; therefore, prolonging antiviral therapy beyond the point at which no CMV DNA is detected in the plasma, is reasonable for optimal treatment. The decision regarding secondary prophylaxis varies [9].

**Figure 2:**
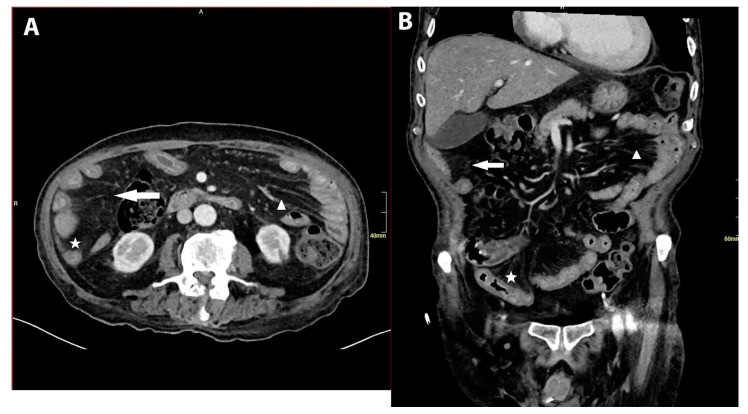
(**A**,**B**): Abdominal CT with signs of infectious enteritis. Axial (**A**) and coronal (**B**) contrast-enhanced CT images showed signs of an infectious enteritis. The capture shows diffused small bowel wall thickening with mucosal hyperenhancement (asterisk) and perienteric fat stranding (arrow). It also shows mesenteric vessel engorgement (arrowhead). The small bowel was affected entirely from the duodenum to the ileocecal valve. We did not confirm ischemic colitis. Conclusion: CMV infection is usually considered in patients with AIDS, hematological malignancies or after tissue and organ transplantation; however, tissue-invasive CMV disease can also appear in an immunocompromised patient due to long-term corticosteroid therapy.

## Data Availability

The datasets used and/or analyzed during the current study are available from the corresponding author upon reasonable request.
